# Kinlessness at Death: Examining End-of-Life Outcomes With Danish Registry Data

**DOI:** 10.1093/geroni/igaa057.2036

**Published:** 2020-12-16

**Authors:** Christine Mair, Katherine Ornstein, Melissa Aldridge, Lau Thygesen

**Affiliations:** 1 University of Maryland, Baltimore County, Baltimore, Maryland, United States; 2 Icahn School of Medicine at Mount Sinai, New York, New York, United States; 3 University of Southern Denmark, Copenhagen, Hovedstaden, Denmark

## Abstract

Demographic changes that lead to “kinlessness” such as low fertility and low marriage rates are not a recent phenomenon for the countries of Northern Europe, such as Denmark. Characterized by small family sizes, high individualism, and a highly formalized healthcare system that is less dependent on family caregivers, Denmark presents a useful case study for the analysis of end-of-life outcomes among the “kinless.” We analyze the population of decedents aged 50 and older (N=175,755) using Danish civil registry data. Approximately 15% of those who died in Denmark had no living partner and no living child. Danish decedents’ family structures are associated with multiple end-of-life outcomes, including number of hospitalizations, ICU visits, and use of specific medical treatments—but not always in the direction hypothesized. Denmark’s highly formalized and individualized healthcare system may offer insight regarding healthcare reform in countries that have yet to complete the second demographic transition.

